# Trends and Factors Influencing Nursing Home Closures: A Retrospective Analysis (1990-2024)

**DOI:** 10.1093/geroni/igaf122.1016

**Published:** 2025-12-31

**Authors:** Joohyun Chung, Michael Lepore, Tingzhong (Michelle) Xue

**Affiliations:** University of Massachusetts Amherst, Amherst, Massachusetts, United States; University of Massachusetts Amherst, Amherst, Massachusetts, United States; University of Massachusetts Amherst, Amherst, Massachusetts, United States

## Abstract

Nursing home (NH) closures have significant implications for access to long-term care, particularly for vulnerable populations reliant on public healthcare programs. This study aimed to compare the characteristics of NHs that closed versus those that remained open from 1990 to 2024. This study explores the geographic distribution, regional variations, and bed capacity of nursing home closures. An observational, quantitative, cross-sectional study design was used. De-identified data from the Centers for Medicare & Medicaid Services (CMS) Provider of Services File were analyzed. Descriptive statistics, Chi-square tests, generalized linear models (GLM), and linear regression were employed to identify trends in NH closures. A total of 114,882 records were retrieved, with 23,903 selected for Skilled Nursing Facility and Nursing Facility types. From 1990 to 2024, there were 23903 total, 14834 active NHs and 9,069 closures. Active NHs had an average of 107 beds, while closed NHs had 70 beds (p < 0.001). Twelve percent of active NHs (N = 1,928) had at least one ADRD bed, compared to 4.7% of closed NHs (N = 433) (p < 0.001). Ninety-four percent of active NHs participated in both Medicare and Medicaid, whereas only 55% of closed NHs did, with 17% participating only in Medicare and 28% in Medicaid. Smaller nursing homes with fewer specialized beds and lower Medicare/Medicaid participation are more likely to close. These findings suggest that changes in Medicare and Medicaid policies could affect the sustainability of resource-limited facilities, highlighting the need for targeted support to maintain access to long-term care services.

